# Whole-genome CNV analysis: advances in computational approaches

**DOI:** 10.3389/fgene.2015.00138

**Published:** 2015-04-13

**Authors:** Mehdi Pirooznia, Fernando S. Goes, Peter P. Zandi

**Affiliations:** ^1^Mood Disorders Center, Department of Psychiatry and Behavioral Sciences, School of Medicine, Johns Hopkins UniversityBaltimore, MD, USA; ^2^Department of Mental Health, Johns Hopkins Bloomberg School of Public HealthBaltimore, MD, USA USA

**Keywords:** whole-genome sequencing, copy number variation, CNVs, computational modeling, structural variation (SV), next generation sequencing

## Abstract

Accumulating evidence indicates that DNA copy number variation (CNV) is likely to make a significant contribution to human diversity and also play an important role in disease susceptibility. Recent advances in genome sequencing technologies have enabled the characterization of a variety of genomic features, including CNVs. This has led to the development of several bioinformatics approaches to detect CNVs from next-generation sequencing data. Here, we review recent advances in CNV detection from whole genome sequencing. We discuss the informatics approaches and current computational tools that have been developed as well as their strengths and limitations. This review will assist researchers and analysts in choosing the most suitable tools for CNV analysis as well as provide suggestions for new directions in future development.

## Background

Rapid advances in genomic technologies over the past decade have revealed that CNVs makes an important contribution to genetic variation in the human genome (Iafrate et al., 2004; Sebat et al., 2004; Macdonald et al., 2014) and plays a role in an increasing number of human diseases, such as autism (Pinto et al., 2010; Chung et al., 2014), schizophrenia (Castellani et al., 2014), major depressive disorder (O’Dushlaine et al., 2014), epilepsy (Olson et al., 2014), and many others (Tan et al., 2014). CNVs refer to a type of structural variation with abnormal copy number changes involving DNA fragments that are typically longer than 1 Kb and results in gains (duplication or insertional transpositions), losses (deletion), or complex rearrangements of the genome (Iafrate et al., 2004; Feuk et al., 2006). On average, each individual has more than 1000 CNVs across the genome, which accounts for ∼4 million bp (Conrad et al., 2010; Malhotra and Sebat, 2012; Abel and Duncavage, 2013). CNVs can involve one or multiple genes and can present as a recessive or dominant allele that disrupts the coding region or alters gene dosage (Zhou et al., 2011). CNVs can also negatively impact the regulatory landscape by generating chimeric genes or by introducing positional effects (Cook and Scherer, 2008; Chung et al., 2014).

Current NGS technologies generate billions of bases of accurate nucleotide sequences in short reads (50–250 bp) using reversible sequencing chemistries (Bentley et al., 2008; Mardis, 2013) rapidly expanding our ability to interrogate the genome. Several new tools have been developed to enable discovery of CNVs from NGS data (Zhao et al., 2013). Each of these tools have different strengths and weaknesses in their applicability and suitability for NGS data, and no single tool is capable of identifying the full range of DNA variation. Comparisons and evaluation of such tools are beginning to emerge (Alkan et al., 2011; Abel and Duncavage, 2013; Zhao et al., 2013; Alkodsi et al., 2014; Tan et al., 2014). Here, we summarize the recent developments in the most widely used CNV detection tools with specific focus on whole genome sequencing data, with the goal of aiding researchers in choosing the most suitable tools for their research needs.

## Methods for CNV Detection

**FIGURE 1:**
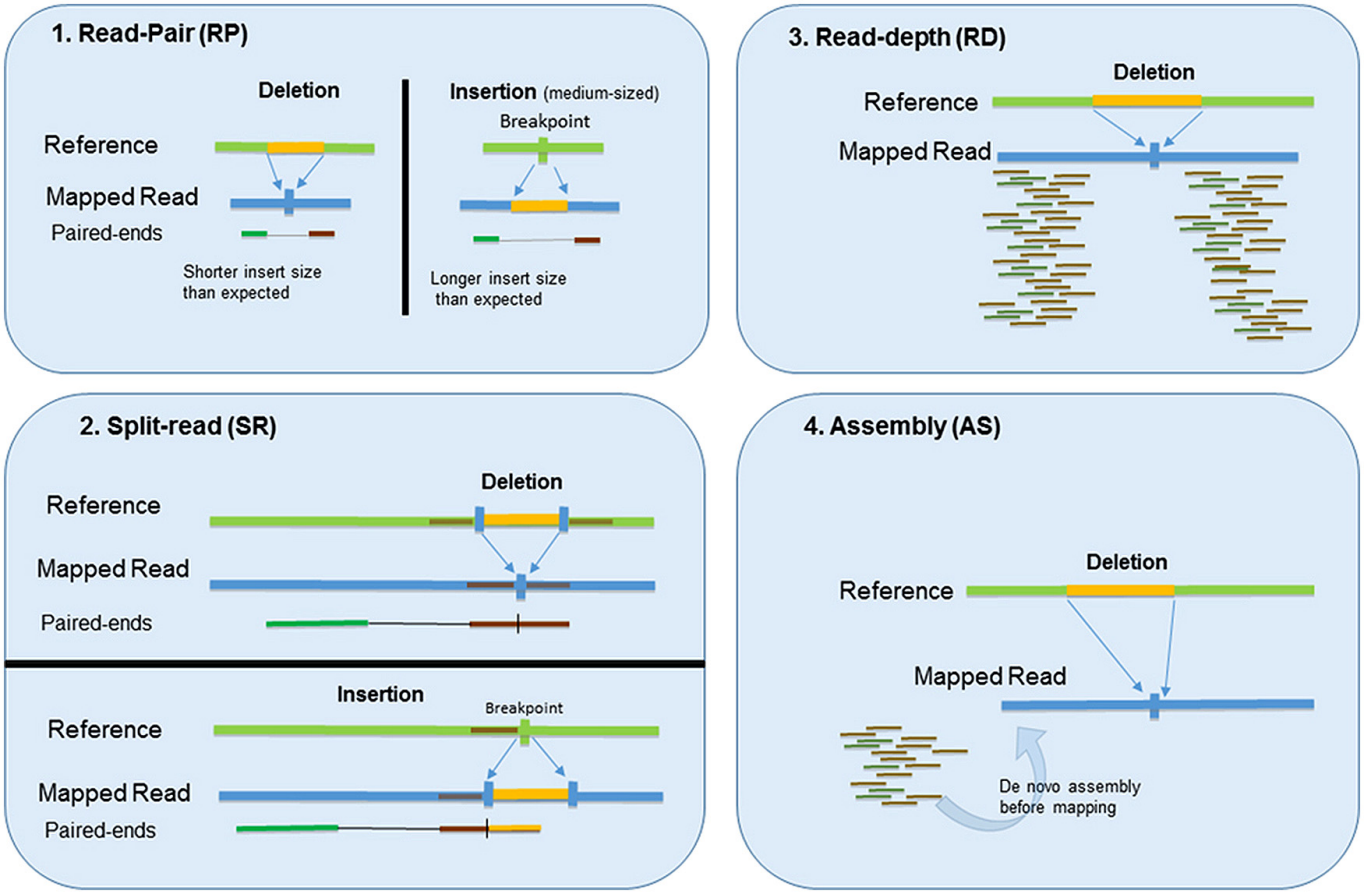
**Four main methods for detecting CNVs with NGS data: (1) Read-pair (RP), (2) Split-read (SR), (3) Read-depth (RD), and (4) Assembly based (AS) method**.

There are four main methods for detecting CNVs with NGS data: RP, SR, RD, and assembly based (AS) methods (Alkan et al., 2009; Medvedev et al., 2009; Yoon et al., 2009; Xi et al., 2012; Duan et al., 2013; Liu et al., 2013; Zhao et al., 2013; Tan et al., 2014). The schemas illustrated in **Figure 1**. Each method has its own advantages and limitations. To take advantage of the method’s different strengths, more recent tools are resorting to a combinatorial approach (Hormozdiari et al., 2009; Zhao et al., 2013) that combines two or more methods to facilitate more accurate CNV detection. We will discuss these four methods and common applications of each in this section. A brief summary of these methods and related tools are listed in **Table 1**.

**Table 1:** **Copy number variation detection analysis tools for WGS data**.

Name	Language	Reference	Availability
**RP: Read Paired**
BreakDancer	Perl/C++	Chen et al. (2009)	http://gmt.genome.wustl.edu/packages/breakdancer/
PEMer	Python/Perl	Korbel et al. (2009)	http://sv.gersteinlab.org/pemer/
Ulysses	Python/R	Gillet-Markowska et al. (2014)	https://github.com/gillet/ulysses
**SR: Spit Read**
PRISM	C	Jiang et al. (2012)	http://compbio.cs.toronto.edu/prism/
Gustaf	C++	Trappe et al. (2014)	http://www.seqan.de/projects/gustaf/
SVseq2	C++	Zhang et al. (2012)	http://www.engr.uconn.edu/~jiz08001/svseq2.html
Pindel	C++	Ye et al. (2009)	http://gmt.genome.wustl.edu/packages/pindel/
**RD: Read Depth**
BIC-seq	Perl/R	Xi et al. (2011)	http://compbio.med.harvard.edu/Supplements/PNAS11.html
cm.MOPS	R	Klambauer et al. (2012)	http://www.bioinf.jku.at/software/cnmops/
CNVnator	C++	Abyzov et al. (2011)	http://sv.gersteinlab.org/cnvnator/
CNV-seq	Perl/R	Xie and Tammi (2009)	http://tiger.dbs.nus.edu.sg/CNV-seq/
CNVrd2	R	Nguyen et al. (2014)	http://www.utahresearch.org/mingfuzhu/erds/
ERDS	C	Zhu et al. (2012)	http://www.utahresearch.org/mingfuzhu/erds/
RDXplorer	Python/R	Yoon et al. (2009)	http://rdxplorer.sourceforge.net/
ReadDepth	R	Miller et al. (2011)	https://github.com/chrisamiller/readDepth
SegSeq	MatLab	Chiang et al. (2009)	http://www.broadinstitute.org/cgi-bin/cancer/publications/pub_paper.cgi?mode=view&paper_id=182
**AS: Assembly**
Magnolya	Python	Nijkamp et al. (2012)	http://bioinformatics.tudelft.nl/dbl/software
**CA: Combined Approach**
cnvHiTSeq	Java	Bellos et al. (2012)	http://sourceforge.net/projects/cnvhitseq/
CNVer	C++	Medvedev et al. (2010)	http://compbio.cs.toronto.edu/CNVer/
Clever-sv	C++	Marschall et al. (2013)	https://code.google.com/p/clever-sv
DELLY	C++/R	Rausch et al. (2012)	https://github.com/tobiasrausch/delly
Gindel	C++	Chu et al. (2014)	http://sourceforge.net/projects/gindel
Hydra-Multi	C++	Lindberg et al. (2014)	https://github.com/arq5x/Hydra
GenomeSTRiP	Java/R	Handsaker et al. (2011)	http://www.broadinstitute.org/software/genomestrip/
GASVPro	C++	Sindi et al. (2012)	http://compbio.cs.brown.edu/projects/gasv/
LUMPY	C++	Layer et al. (2014)	https://github.com/arq5x/lumpy-sv
PSCC	Perl	Li et al. (2014)	http://public.genomics.org.cn/BGI/PSCC/
SoftSearch	Perl	Hart et al. (2013)	https://code.google.com/p/softsearch
SV Detect	Perl	Zeitouni et al. (2010)	http://svdetect.sourceforge.net/Site/Home.html

### Read-Pair

The utility of NGS data for CNV detection was first demonstrated by RP methods. RP methods compare the average insert size between the actual sequenced read-pairs with the expected size based on a reference genome. In paired-end sequencing, the DNA fragments are expected to have a specific distribution around insert size (Korbel et al., 2007). As such, the discordance between mapped paired-reads whose distances are significantly different from the predetermined average insert size is utilized by RP to identify CNVs. While RP methods can detect medium-sized insertions and deletions from mapped data, they are insensitive to small insertion, or deletion events, owing to the difficulty in separating small perturbations in read-pair distance from the normal background variability (Medvedev et al., 2009). Furthermore, RP methods are not applicable for detection of CNVs in low-complexity regions with segmental duplication (Zhao et al., 2013). Tools that use the RP method include PEMer, Hydra, Ulysses, and BreakDancer. The relative advantages and limitations of these methods are briefly discussed below.

#### PEMer

PEMer (Korbel et al., 2009) utilizes a clustering based strategy to detect CNVs and is applicable to several different next-generation DNA sequencing platforms, including Roche, Illumina, and ABI. The clustering step combines paired ends that are likely originated from the same SV into clusters. It also evaluates different parameterizations, by applying different cluster sizes and cutoffs for outlier identification. Owing to its modularized framework such as mapping, filtering of low-quality reads, signature detection, and clustering, PEMer offers the feasibility of amending particular modules to suite the user’s needs without having to implement an entirely new SV discovery pipeline and thus improvise an existing pipeline.

#### Ulysses

Ulysses (Gillet-Markowska et al., 2014) allows an accurate detection of low-frequency CNVs in large insert-size sequencing libraries (Mate–Pair libraries) providing higher coverage of the genome and thereby access the repeat-containing regions. It uses statistics based on the relative coverage of candidate SVs to achieve higher specificity.

#### BreakDancer

BreakDancer (Chen et al., 2009) contains two complementary algorithms: BreakDancerMax and BreakDancerMini. BreakDancerMini uses a model-based Kolmogorov–Smirnov test as a mapping algorithm and detects smaller indels (10–100 bps) while BreakDancerMax uses a clustering-based approach and reports deletions, insertions, inversions, and intra and inter-chromosomal translocations. One limitation of BreakDancer is that it only uses unique mapped reads and discards reads with multiple mapping and therefore is not able to detect CNVs in low complexity repetitive regions.

### Split Read

Split Read method uses reads from pair end sequencing where only one read of the pair has a reliable mapping and the other one either completely or partially fails to map to the genome (Zhang et al., 2011). The unmapped reads are a potential source of breakpoints at the single base pair level. Mapping of reads that span across a breakpoint of an SV provides the precise start and end positions of the segments that are INDEL events. Split-read based methods, including Pindel, Gustaf, SVseq2, and Prism, while able to identify these breakpoints, have limited ability to identify large-scale SVs. Prism, however, seems to substantially overcome this limitation by employing a modified Needleman–Wunsch alignment algorithm (Jiang et al., 2012).

#### Pindel

Pindel (Ye et al., 2009) uses *de novo* alignment of the unmapped reads to determine the exact sequence of an insertion, and therefore is capable of identifying break points of medium or large-sized insertion of paired-end short reads that might be ignored by other tools. However, since it does not use probabilistic models to discriminate between alignment errors and true calls, a higher false-positive rate can be observed (Abel and Duncavage, 2013).

#### Gustaf

Gustaf (Trappe et al., 2014) is based on multi-split SV detection tool that detects all classes of SVs that are ≥30 bp length. The multi-split alignment strategy can identify SV breakpoints with base pair resolution. Gustaf uses the local aligner to detect partial alignments of a read and stores these partial alignments in a graph data structure so it can be used in the subsequent split-graph construction. This feature gives Gustaf the ability to detect SVs that are hard to classify including dispersed duplications and translocations.

#### PRISM

Prism (Jiang et al., 2012) makes use of discordant pair-end clusters to perform split-read mapping. The modified Needleman–Wunsch (NW) algorithm provides better performance for the base-level alignment of the SPs to achieve higher accuracy when other variations (SNPs, Indels) exist. These functionalities lead to faster run times as well as higher sensitivities at detection of large CNVs.

#### SVseq2

SVseq2 (Zhang et al., 2012) supports INDEL calling from low-coverage sequence data. SVseq2 infers a focal region, using the discordant read analysis. It then searches for the occurrence of the second segment within the focal region using a semi-global alignment algorithm, which can lead to more accurate SV calls.

### Read Depth (RD)

As the name implies, RD methods are based on the hypothesis that there is a correlation between depth of coverage of a genomic region and the copy number of the region (Teo et al., 2012). Based on the study design, RD methods can be categorized into three classes; single sample, paired case/control samples, and a large population of samples. In the single sample category, as there is no other subject available, the absolute copy number will be reported; in presence of controls, the relative copies compared to controls will be reported; and, in population based studies, the overall mean of the RD will be used to detect CNVs (Zhao et al., 2013). Compared to RP and SR, RD can detect the exact number of CNVs, as RP, and SR can only report the position of the potential CNVs and not the counts. In addition, RD can work better on large size CNVs, which are hard to detect with RP and SR (Yoon et al., 2009). Estimating CNVs using RD method follows these steps. First reads are aligned to a reference genome and RD will be counted using a predefined window. Then the counts will be normalized to remove potential biases, mainly due to GC content and repeat regions (Boeva et al., 2011; Janevski et al., 2012), and a segmentation algorithm will be applied to identify a contiguous set of windows having the same number of CNVs. Finally, the statistical significance of the calls will be predicted and filtering will be applied (Janevski et al., 2012; Zhao et al., 2013).

#### CNV-seq

CNV-seq (Xie and Tammi, 2009) uses the read coverage of the data and calculates the best window size in which copy ratios between the case and control are significantly different. Using this window size its algorithm models the number of short reads in a genomic region as following a Poisson distribution. The Poisson distribution, however, might not be an optimal model in many CNVs, and therefore, more sophisticated models might be required (Xi et al., 2010).

#### BIC-seq

BIC-seq (Xi et al., 2011) uses a non-parametric model for detecting CNVs from paired sequencing data. It uses a heuristic greedy search procedure which is a more computationally efficient strategy compared to other tools.

#### Cm.MOPS

cm.MOPS (Copy number estimation by a Mixture Of PoissonS; Klambauer et al., 2012) is a CNV detection pipeline that models the depths of coverage across multiple samples at each genomic position. Using a Bayesian approach, it decomposes read variations across samples into integer CNVs and noise using mixture components and Poisson distributions, respectively. The multiple samples approach increases statistical power and decrease computational burden and the FDR in CNV detection.

#### CNVnator

CNVnator (Abyzov et al., 2011) uses the established mean-shift approach (Wang et al., 2009) with additional corrections for multiple-bandwidth partitioning and GC correction for more accurate CNV detection. It is capable of detecting CNVs in various sizes, from a 500 bp window for 4–6× coverage, to a 30 bp window for 100× coverage.

#### ERDS

ERDS (Estimation by Read Depth with SNVs; Zhu et al., 2012) integrates RD with other information including paired end mapping and soft-clip signature, as well as GC correction and employing HMM at non-amplified regions to achieve more sensitive and accurate CNVs. The soft-clipping process masks the unaligned portion of a read and try to re-map it unambiguously to a different genomic location, to identifying the second breakpoint for a potential SV (Wang et al., 2011).

#### RDXplorer

RDXplorer (Yoon et al., 2009) is based on the event-wise testing (EWT) algorithm and estimates CNVs in a non-overlapping intervals (100 bp Windows) across an individual genome. The EWT algorithm rapidly searches the entire genome for specific classes of small events (significantly increased or reduced RD) that meet criteria of statistical significance, and then clusters them into larger events. A–Z-score is then calculated based on the number of reads mapped in each 100 bp window.

#### ReadDepth

ReadDepth (Miller et al., 2011) automatically sets an appropriate size for a sliding window according to the mean number of reads in each window. It does not require a reference sample, and uses a robust statistical model that uses a negative-binomial distribution to approximate an overdispersed Poisson distribution of the data. It also includes multi-core architectures to parallelize the analysis process effectively, and it increases the resolution obtained from low-coverage experiments using breakpoint information from paired end sequencing to do positional refinement. RD is also capable of discovering epigenetic changes by processing bisulfite-treated reads.

#### SegSeq

SegSeq (Chiang et al., 2009) utilizes windows defined by a predefined number of normal reads to detect breakpoints. It uses the log ratio of the case versus control read counts as the statistic for CNV detection. The latest version of SegSeq can be used for analysis of different sequencing depths of 1–30×.

#### CNVrd2

CNVrd2 (Nguyen et al., 2014) first uses observed read-count ratios to refine segmentation results in one population. Then, in the next step, it applies a linear regression model to adjust the segmentation scores between populations and uses a Bayesian normal mixture model to cluster segmentation scores into groups for individual CNV counts.

### Assembly (AS)

In theory, all forms of genetic variation including CNVs can be detected by AS of short reads, if the reads are long and accurate enough. The AS methods first generate a contig/scaffold that are then compared with the reference genome to discover structural variation (Nijkamp et al., 2012; Teo et al., 2012). However, AS methods are less used in CNV detection due to their overwhelming demand on computational resources. In addition, eukaryotic genomes contain a significant fraction of repeats and segmental duplications which makes the AS methods less accurate and more complex as they perform poorly in these complex regions. Another issue with the AS methods is that they are unable to handle haplotype sequences and therefore only homozygous structure variations can be detected (Xi et al., 2012).

#### Magnolya

Magnolya (Nijkamp et al., 2012) estimates CNVs from two or more samples by utilizing a Poisson mixture model of contigs assembled from sequencing data. This will be followed with a co-assembly approach to allow *de novo* detection of CNV between two individual genomes. The co-assembly approach generates a single contig colored graph with different counts between samples which will be used to assign integer copy numbers to contigs.

### Combined Approach (CA)

Each of the methods mentioned above has its own strengths and limitations. While RD based methods are best suited for detecting absolute copy number (Alkan et al., 2009) they suffer from lower efficiency for determining small CNVs (<1 kb; Bellos et al., 2012). Tools using RP, on the other hand, have low sensitivity for detecting variation in repeating regions (Medvedev et al., 2009). This seems to be the same issue with SR approaches as they can achieve single-base-pair resolution but remain highly dependent on the read length and are less reliable in repetitive regions (Bellos et al., 2012). AS-based tools take advantage of not requiring a reference genome, but they suffer from extensive computation and perform poorly on repeat regions (Zhao et al., 2013). CA methods use step-wise approaches to combine data from two or more sources. In doing so, CA methods take advantage of the unique features of multiple tools. For instance, RP methods can report accurate breakpoints although their efficiency is low for large CNV regions when detecting insertions longer than the insert size. RD methods, on the other hand, are more suited to detect large CNVS but cannot report exact breakpoints. A combination of these two methods would in essence enable detection of CNV regions with exact breakpoints and spanning various length. As such, the false positive rates observed with CA methods are much lower than methods that build on either RP or RD alone. Currently, several tools that utilize CA to identify CNVs are available. They are briefly discussed below.

#### SVDetect

SVDetect (Zeitouni et al., 2010) is one of the first tools that combined an RP approach and RD ratios between case and control samples. It uses the discordant RP information to identify breakpoints and RD signals to identify aberrant genomic fragments.

#### cnvHiTSeq

cnvHiTSeq (Bellos et al., 2012) uses an integrative approach by combining outcomes from RD, RP, and SR to detect all CNV classes even from low-coverage sequence data. It implements an HMM framework to perform CNV segmentation. In addition, it utilizes LOESS smoothing and GC correction to mitigate sequencing biases.

#### Clever-sv

Clever-sv (Marschall et al., 2013) combines SR and discordant RP reads to call CNVs. It works best for calling genotypes of midsize deletions at medium coverage.

#### CNVer

CNVer (Medvedev et al., 2010) combines RP and RD information for CNV detection. It also implements an ambiguous mapping strategy which does not rely on having uniquely best read mappings, and uses all good mappings for every mate pair which can result in higher sensitivities in repeat and duplication regions.

#### DELLY

DELLY (Rausch et al., 2012) analyzes discordant RP first and then attempts to strengthen the results with supporting SR. It enables to ascertain the full spectrum of genomic rearrangements, including CNV events as well as balanced rearrangements.

#### GenomeSTRiP

GenomeSTRiP (Haraksingh and Snyder, 2013) combines several sources of information contained in the sequence reads, including discordant RP as a starting point and RD as a downstream filter. It can accurately call genotypes of relatively long CNVs (≥200 bp). It is capable of working with large populations (Genomes Project et al., 2010) and works best when data from at least 20 individuals is analyzed together.

#### Gindel

Gindel (Chu et al., 2014) uses a support vector machine (SVM) learning approach which combines multiple features extracted from NGS data. These features include discordant RP, SR, spanning reads (reads mapped to a region that overlaps the indel), and RD near the deletion.

#### GASVPro

GASVPro (Sindi et al., 2012) integrates RP and RD methods via a Markov Chain Monte Carlo probabilistic model to achieve improved specificity in detection of structural variation especially in repetitive regions.

#### Hydra-Multi

Hydra-Multi (Lindberg et al., 2014) works with multi-sample to detect SVs. Its algorithm begins by routing discordant alignments from RP, followed by identifying candidate breakpoint clusters via an efficient sorting strategy, and concludes with an AS method to implement a greedy breakpoint reconstruction. Its sorting algorithm enables multi-sample SV analysis of hundreds of genomes in a very efficient way.

#### LUMPY

LUMPY (Layer et al., 2014) is a probabilistic model integrating in parallel any or all of the three different signals, RP, SR, and RD, from a genome single sample. In addition, LUMPY can incorporate sites of known variants, if provided, as a prior knowledge in order to improve sensitivity.

#### PSCC

PSCC (population-scale CNV calling; Li et al., 2014) combines RP and RD. It uses a two-step correction procedure (self-adjustment with GC content and population based normalization) to remove biases caused by local GC content and complex genomic characteristics. PSCC uses a binary segmentation method to locate CNV segments and a combined statistics test to ensure the best performance with regard to false positive control, resulting in improved specificity.

#### SoftSearch

SoftSearch (Hart et al., 2013) utilizes SR and RP strategies for detecting SVs to increase sensitivity. It first identifies areas with soft-clipping (Co-localized SR method) in the genome with discordant read pair information, then it extracts the read and mate information directly from the aligned file, resulting in a fast, and consistent run time with high sensitivity.

## Considerations

### Method Limitations

In recent years, the CA has become more popular due to the fact that none of the four methods by themselves are sufficiently comprehensive. There are pros and cons to each method with regards to CNV detection depending on the underlying structure at the SV site (Alkan et al., 2011). SR can detect the exact breakpoints of SVs. However, it is limited to the length of the reads and NGS data shorter than 1 kb affect the accuracy and precision. In addition, SR is currently reliable only in the unique regions of the genome (Zhang et al., 2011). RP is able to identify almost all types of SVs, but it is unable to detect the exact breakpoints with loose fragment size distributions. The accuracy of RP methods is largely dependent on the insert size. While small events can be missed with large-insert libraries, insertions larger than the library insert size might be ignored (Le Scouarnec and Gribble, 2012). Both RP and SR methods have poor performance in regions enriched with duplications since they rely on confident and independent mapping of each end (Yoon et al., 2009; Li and Olivier, 2013). AS generates a long sequence from the short reads, called contig/scaffold, that match the reference genome. However, it has been shown that AS has a poor performance against duplications or repeats (Alkan et al., 2011). In the RP approach, resolving ambiguous mappings in repetitive regions is challenging and accurate prediction of SV breakpoints depends on fragment size distributions, which can result in costly and complicated library construction (Medvedev et al., 2009). RD is more reliable for regions with deletions and duplications and can also count the number of CNVs. However, similar to RP, it is difficult to identify the exact breakpoints in RD. Compared with RP, it is anticipated that RD events are enriched in segmental duplications (Yoon et al., 2009). Although RD is the only method to accurately predict absolute copy numbers, the breakpoint resolution is often poor (Alkan et al., 2009). All of the limitations outlined above can result in discovery of only a subset of SV/CNVs. This has prompted the recent development of algorithms that integrate multiple methods to improve sensitivity and specificity (Kidd et al., 2010; Alkan et al., 2011; Zhao et al., 2013).

### Mappability and Coverage

In the RD approach the read alignment can introduce potential bias. A significant number of reads may not be mapped uniquely or mapped to multiple positions due to the presence of repetitive regions in the reference genome and short read length of the NGS technology. RD based methods that ignore these multiple aligned reads (Chiang et al., 2009; Xi et al., 2011) perform poorly in homologous genomic regions. Methods that assign an ambiguous read to one of all possible positions perform better than those using only uniquely mapped reads, but with higher false positives (Abyzov et al., 2011). Integrated CA approaches such as Hydra, ERDS, and SoftSearch mitigate this issue by employing a soft clustering approach that improves the CNV detection sensitivity.

### CNV Size and Distribution

Read depth-based methods uses a fixed window approach to infer RD signal from reads to identify potential SVs. Larger windows can achieve higher confidence for CNV calls. However, small CNVs are easy to miss and difficult for RD methods to detect. Approaches that combine RP with RD and AS can improve both sensitivity and specificity for CNV detection for small CNVs (Tan et al., 2014). The library insert size is also an important aspect of the CNV detection. Long insert sizes have the advantage of detecting larger events with higher confidence, while shorter insert sizes increase the sensitivity for smaller events (Medvedev et al., 2009).

### GC bias Normalization and Control Samples

Algorithms such as EWT that do not require control data, detect CNVs by calculating deviations in coverage depth from the sample’s mean depth (Yoon et al., 2009). However, many factors, such as GC content, affect the coverage profile, and therefore these biases must be corrected to provide adequate specificity (Benjamini and Speed, 2012). GC content varies along the genome and has been found to influence read coverage on most sequencing platforms. In general, regions with low or high GC content have low depth of coverage (Teo et al., 2012). Methods designed for case-control comparisons avoid this issue by matching the same region across multiple samples by partitioning the genome into regions, calculating the depth of coverage ratio between case and control for each region, and then partitioning the region into segments of equal copy number, using a variety of approaches, including HMMs (Chiang et al., 2009; Xie and Tammi, 2009; Ivakhno et al., 2010). These algorithms, because they rely on the coverage ratio rather than the raw coverage profile, permit finer mapping of CNV boundaries using, for instance, mean-shift approaches from signal processing (Abyzov et al., 2011).

## Conclusion and Future Directions

Although NGS has led to marked improvement in the detection of structural variation, accurate detection of CNVs in a computationally feasible manner continues to be a challenge. The field still lacks a single informatics method that is applicable to wide variety of structural DNA variations. Recent studies have also observed inconsistencies in the output from different platforms and analysis methods (Alkan et al., 2011; Alkodsi et al., 2014; Tan et al., 2014). Consequently, a CA has proven more effective in addressing the inadequacy of the different methods/tools and has led to variant detection with improved sensitivity and reliability. However, the development of standard protocols, quality control, and benchmarking, and extensive laboratory validations is required in order to calibrate existing CNV analytical tools and foster the development of new algorithms for the next-generation of sequencing technologies.

## Conflict of Interest Statement

The authors declare that the research was conducted in the absence of any commercial or financial relationships that could be construed as a potential conflict of interest.
